# Isomerization and Stabilization of Amygdalin from Peach Kernels

**DOI:** 10.3390/molecules28114550

**Published:** 2023-06-05

**Authors:** Decai Zhang, Jianfen Ye, Yu Song, Yingying Wei, Shu Jiang, Yi Chen, Xingfeng Shao

**Affiliations:** College of Food and Pharmaceutical Sciences, Zhejiang-Malaysia Joint Research Laboratory for Agricultural Product Processing and Nutrition, Ningbo University, Ningbo 315800, China; nbuzdc8010@163.com (D.Z.); yejianfen@nbu.edu.cn (J.Y.); cxhtzxsy@126.com (Y.S.); weiyingying@nbu.edu.cn (Y.W.); jiangshu@nbu.edu.cn (S.J.); chenyi@nbu.edu.cn (Y.C.)

**Keywords:** amygdalin, isomerization, HepG2 cells, peach kernels, hydrogel beads

## Abstract

In this study, isomerization conditions, cytotoxic activity, and stabilization of amygdalin from peach kernels were analyzed. Temperatures greater than 40 °C and pHs above 9.0 resulted in a quickly increasing isomer ratio (L-amygdalin/D-amygdalin). At acidic pHs, isomerization was significantly inhibited, even at high temperature. Ethanol inhibited isomerization; the isomer rate decreased with the ethanol concentration increasing. The growth-inhibitory effect on HepG2 cells of D-amygdalin was diminished as the isomer ratio increased, indicating that isomerization reduces the pharmacological activity of D-amygdalin. Extracting amygdalin from peach kernels by ultrasonic power at 432 W and 40 °C in 80% ethanol resulted in a 1.76% yield of amygdalin with a 0.04 isomer ratio. Hydrogel beads prepared by 2% sodium alginate successfully encapsulated the amygdalin, and its encapsulation efficiency and drug loading rate reached 85.93% and 19.21%, respectively. The thermal stability of amygdalin encapsulated in hydrogel beads was significantly improved and reached a slow-release effect in in vitro digestion. This study provides guidance for the processing and storage of amygdalin.

## 1. Introduction

Amygdalin is a cyanogenic glycoside, a derivative of α-hydroxy nitrile, and a simple natural chemical that is widely found in the seeds of many Rosaceae [1,2]. It is a glycoside compound which is composed of two glucose and one nitrile; its molecular formula is C_20_H_27_O_11_N; and its molecular weight is 457.43 g/mol. D-amygdalin has pharmacological activity; it has been used clinically for its anti-tussive, anti-asthmatic, anti-tumor, anticoagulant, and anti-inflammatory properties, to name a few [3]. It is reported in Li et al. [4] that amygdalin can induce apoptosis of breast cancer cells and inhibit the adhesion of breast cancer cells was confirmed. Furthermore, antiapoptotic protein Bcl-2 was downregulated and proapoptotic Bax protein was upregulated in HeLa cells after treatment by amygdalin [5].

There are two forms of amygdalin: L- and D-amygdalin (Supplementary Figure S1); naturally occurring amygdalin is only in the D-confirmation. At high temperatures or in and alkaline environment [6,7], D-amygdalin isomerizes into the L-form. L- and D-amygdalin are epimers, not enantiomers; their glucosidal unit centers differ in configuration with only one stereocenter [8]. Although isomers are identical in molecular composition and weight, there can be huge differences in biological activity. As reported by Song et al. [9], who investigated the growth inhibition activity of different ratios of L/D-amygdalin on PC12 and MDCK cells, the greater the proportion of D-amygdalin, the greater the inhibition of growth. As much as current research has focused on D-amygdalin, there is little information concerning the distinction between D- and L-amygdalin, particularly regarding the anti-tumor activity of L-amygdalin. The isomerization of amygdalin is influenced by many factors, such as temperature, pH, solvent, and container material. A. Nahrstedt [7] found that D-amygdalin could be converted to L-amygdalin in boiling water, and that the proportion of L-amygdalin increased with the prolongation of heating time until the content of L-amygdalin finally stabilized at 53% after 2 h in glass bottle. He also found that heating amygdalin in ethanol (95 °C) converted 14% of the D-amygdalin into the L-amygdalin. It has long been known that in the presence of water and weak bases, epimerization of the amygdalin stereogenic carbon occurs [10]. Wahab et al. [11] noted that container material had a significant effect on the isomerization of amygdalin. They report that Pt and plastic containers significantly inhibit isomerization over glassware. Unfortunately, the research on the mechanism of amygdalin isomerization was still relatively scarce.

Amygdalin is mainly extracted from bitter apricots, semen prunus, almonds, and peach kernels. The extraction methods include reflux extraction, distillation, ultrasonic extraction, hot water, and microwave-assisted extraction [12]. These extraction methods are focused on the yield of amygdalin [13,14]; there are relatively few studies of the isomerization of amygdalin during the extraction processing. In 2017, Xu et al. reported the high content of L-amygdalin in hot water extracts from bitter almonds. Ultrasonic extraction has the unique advantages of short extraction time, high extraction rate, and low extraction temperature, and it is widely used in the extraction of Chinese medicinal materials from plants [15]. Changes of amygdalin and volatile components of apricot kernels during the ultrasonically-accelerated debittering were investigated by Zhang et al. [14], who found that the isomerization of amygdalin was not caused during ultrasonic debittering.

Peach kernels constitute 5–10% of the total fruit weight; they are a by-product of the industrial processing of peach fruit and generally considered waste. Amygdalin accounts for about 1.2–2.4% of peach kernels [16], which is significantly higher than that of other fruit kernels [17]. Xu et al. [18] reported that ethanol-refluxing extraction from peach kernels yielded 87.53% of amygdalin. However, whether amygdalin had isomerized during the extraction process was not reported.

Here, the factors influencing isomerization of amygdalin were investigated to evaluate the differences in cytotoxic activity and the effect of D- and L/D-amygdalin on the growth inhibition of HepG2 cells. The optimized extraction method for obtaining amygdalin from peach kernels was used to compare the yield and isomerization ratio with those of conventional methods. Sodium alginate hydrogel beads loaded with amygdalin are prepared to improve thermal stability of amygdalin and the release effect in in vitro digestion.

## 2. Results and Discussion

### 2.1. Factors Affecting Amygdalin Isomerization

#### 2.1.1. Temperature, pH, Ethanol Concentration, Heating Time, and Container Material

**Figure 1:**
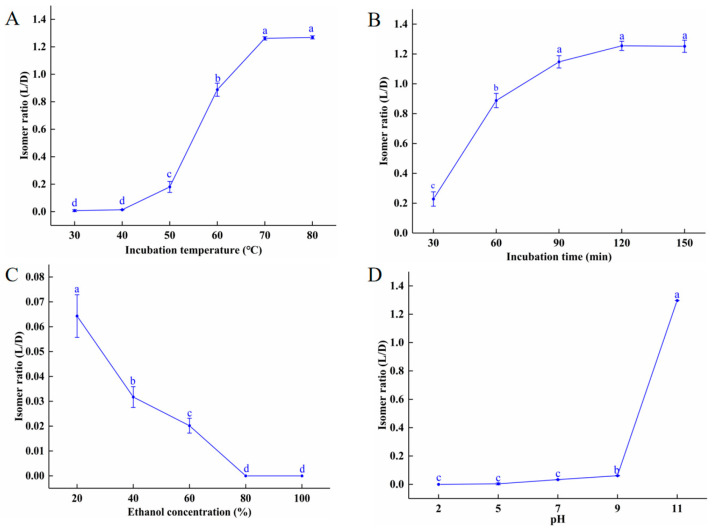
Effects of different factors on isomerization of amygdalin: (**A**) incubation temperature; (**B**) incubation time; (**C**) ethanol concentration; (**D**) pH. Different letters indicate significant differences (*p* < 0.05).

As shown in Figure 1A, there are almost no isomerization at temperatures below 40 °C. From 50 to 70 °C, the L/D isomer ratio increased sharply, reaching its peak (1.27) at 70 °C. There was no further increase in the isomer ratio between 70 and 80 °C. As discussed in Unchiyama et al. [19], high temperatures promote the transformation of chemical compounds, such as catechins and hesperidin, resulting in spatial isomerism. Figure 1B shows that at 60 °C, the isomer ratio increased steadily with incubation times between 30 and 90 min, increasing from 0.23 to 1.2; more than 90 min of incubation did not statistically increase the isomer ratio. These data show that the isomer ratio began increasing steadily at 50 °C and peaked at 70 °C. At 60 °C, incubation times greater than 90 min had no effect on the isomer ratio. As seen in Figure 1C, ethanol had a concentration-dependent inhibitory effect on isomerization, which may be due to the decreasing water content. Turczan [20] and Nahrstedt [7], respectively, found that there was no conversion of D-amygdalin to L-amygdalin in dimethyl sulfoxide or 100% ethanol after 120 min reaction time. We also found that there was no D- to L-amygdalin isomerization when D-amygdalin powder was incubated at 80 ℃ for 2 h (Supplementary Figure S2), indicating that dry D-amygdalin is very stable at high temperatures, and that D to L isomerization requires water molecules.

pH also affects the isomer ratio of amygdalin. As seen in Figure 1D, at acidic, neutral, and slightly basic pHs, no D-amygdalin converted to L-amygdalin at room temperature. At pH 9.0, the isomer ratio was 0.06; at pH 11 the ratio had increased to 1.30. It should be noted that amygdalin hydrolyzes under strong alkaline conditions. Similar results were reported by Seong et al. (2000), who found that under acidic and neutral conditions, amygdalin existed only as D-amygdalin, but under basic conditions, both D- and L-amygdalin were present at a ratio of 1:1.3. It is important to note that under the conditions most conducive to isomerization (80 °C, pH 11), not all D-amygdalin is converted to L-amygdalin; the amygdalin equilibrium constant is *K* = 1.3, with kinetic constants *K*_D_ = 3.4 × 10^−4^ s^−1^ and *K*_L_ = 2.6 × 10^−4^ s^−1^ in deionized water [7]. Thus, it is recognized that the L-epimer would predominate in a mixture. To conclude, acidic and neutral conditions inhibit amygdalin isomerization while alkaline conditions promote it. The promotion of isomerization is due to the benzyl-based protons in the amygdalin molecule which are electron-giving [21].

#### 2.1.2. Anion Type and Concentration in Solvent

**Figure 2:**
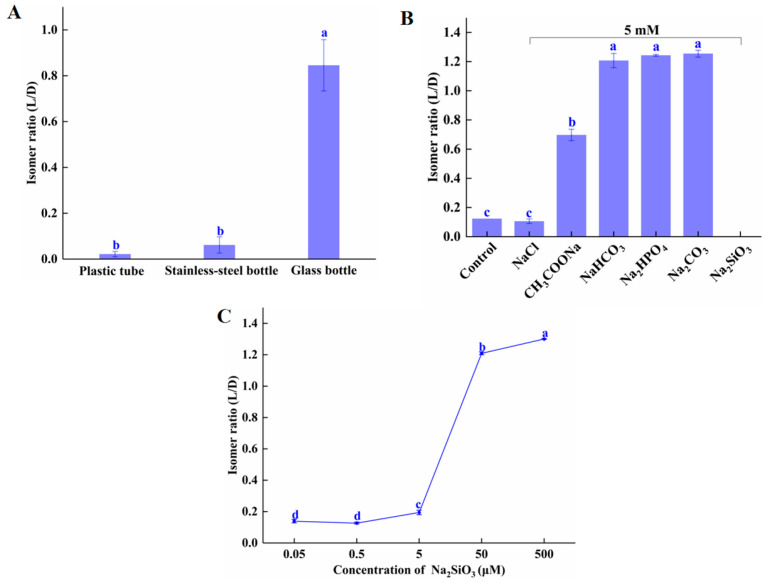
Effect of (**A**) container material (**B**) weak acid ions and (**C**) Na_2_SiO_3_ concentration on amygdalin isomerization. Different letters indicate significant differences (*p* < 0.05). Note: in the Na_2_SiO_3_ treatment group, no amygdalin was detected.

To determine the effect of reaction vessel material on isomerization, we tested the extent of isomerization in glass, plastic, and stainless steel tubes. As shown in Figure 2A, after incubation at 60 °C for 60 min, the L/D isomer ratio of amygdalin in glass was 0.85—significantly higher than in plastic (0.01) and stainless steel (0.02). These results are similar to those reported by Wahab et al. [11], and the hypothesis is that the glass leaches soluble components, such as silicates, which act as weak bases, thus promoting the isomerization of amygdalin. Supplementary Figure S3 shows that the isomer ratio of amygdalin dissolved in water that had been heated in a glass tube was significantly higher than that of amygdalin dissolved in water heated in the plastic tube. As shown in Figure 2B, after incubation at 80 °C for 30 min, the L/D isomer ratios of amygdalin incubated in purified water (control) and 0.01 M NaCl were statistically the same (0.12 and 0.11, respectively). L/D isomer ratios of amygdalin incubated in 0.01 M CH_3_COONa, NaHCO_3_, Na_2_HPO_4_, and Na_2_CO_3_ were 0.70, 1.20, 1.24, and 1.25, respectively. These data show that it is weak acid ions that affect the isomerization of amygdalin, not sodium ions. The weak acid group was ionized by the weak acid group in the water, then the weak acid group was further hydrolyzed to produce OH^−^. The comparative hydrolysis equilibrium constants of the weak acid ions are SiO_3_^2−^ > CO_3_^2−^ > HCO_3_^−^ > H_2_PO_4_^2−^ > CH_3_COO^−^; therefore, the difference in the influence of these weak acid salts on amygdalin isomerization is likely due to their hydrolyzing ability. There was no amygdalin detected in the solution containing Na_2_SiO_3_; possibly it decomposed. This may be caused by the high concentration of weak acid ions in the solution, inducing a mass release of OH^−^. That weak acid ions can promote conformational conversion of substances has been reported previously. In short, weak acid ions promoted isomerization, but high concentrations promoted degradation of amygdalin.

Figure 2C shows that the concentration of Na_2_SiO_3_ is closely related to amygdalin isomerization. The isomer ratio of D-amygdalin incubated with Na_2_SiO_3_ at 5 μM and below was negligible; at 50 μM, the isomer ratio was significantly increased (from 0.195 at 5 μM to 1.209 at 50 μM).

The benzyl-based protons in the amygdalin benzene ring, free OH^−^, or other weakly acid ions (HCO_3_^−^, S^2−^, HSO_3_^−^, ClO^−^, SiO_3_^2−^) in solution with D-amygdalin cause stereoselective isomerization [22,23]. Our and others’ results indicate that glass is not suitable for the storage or processing of amygdalin, especially at high temperatures. Additionally, for solubilizing amygdalin, the concentration of strong base/weak acid salts should be below 5 μM.

#### 2.1.3. Interactions between Factors

**Figure 3:**
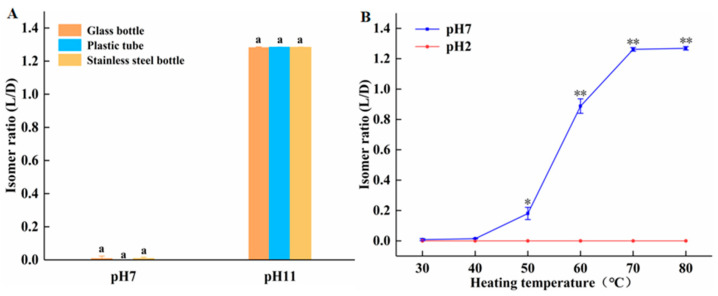
Effect of temperature, pH, and container material on isomerization of amygdalin. (**A**) D-amygdalin was incubated at room temperature in tubes of different materials at neutral and alkaline pH. (**B**) D-amygdalin was incubated at various temperatures in glass tubes at neutral and acid pH. Different letters indicate significant differences (*p* < 0.05). Significance: * *p* < 0.05; ** *p* < 0.01 vs. pH 2.

The interaction of temperature, pH, and container material on amygdalin isomerization is further investigated. As seen from Figure 3A, when incubated at room temperature, the isomer ratio of amygdalin in all containers is very low and statistically identical under neutral conditions, indicating that container material is unimportant under those conditions. At pH 11, the isomer ratio of amygdalin in all containers is very high and statistically identical, indicating that under these conditions, container material is unimportant, but an alkaline pH promotes isomerization. (Figure 3B) Incubating amygdalin in glass tubes at temperatures ranging from 30 to 80 °C at neutral and acidic pHs, the D/L isomer ratio increased with temperature only in the neutral condition. At pH 2, there was no L-amygdalin formed at any temperature. The free H^+^ at pH 2 neutralizes the OH^−^ groups on the amygdalin benzyl ring, rendering the molecule a higher degree of stability. In summary, at room temperature the isomerization of amygdalin only occurred under alkaline conditions, while at elevated temperatures, isomerization could also occur under neutral conditions, though not under acidic conditions.

These data demonstrate that amygdalin should be stored in plastic containers, and that a dry and acidic environment helps prevent isomerization.

### 2.2. The Effect of Amygdalin Isomerization on Cell Viability

#### 2.2.1. Cell Viability and IC_50_

**Figure 4:**
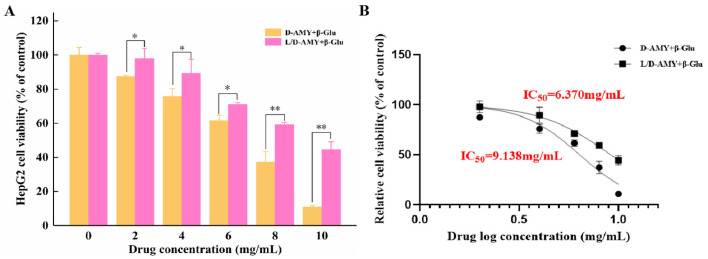
Effect of D- and D/L-amygdalin on (**A**) cell proliferation; (**B**) IC_50_ calculation. Significance: * *p* < 0.05; ** *p* < 0.01.

The cytotoxic effect of D-amygdalin has been reported by numerous researchers [24,25,26]; however, no cytotoxic activity of L-amygdalin has been reported. To identify cytotoxic activity of D and L/D-amygdalin (isomer ratio 1), a MTT assay was used to determine their effect on the growth of HepG2 cells. Although amygdalin itself is not cytotoxic to tumor cells [24], when activated by β-glucosidase, it can inhibit cell proliferation. Β-glucosidase hydrolyzes amygdalin into benzaldehyde and HCN, stimulating the production of ROS which promote apoptosis [27,28]. As seen in Figure 4A, the viability of HepG2 cells incubated for 24 h with D and L/D-amygdalin decreased as the concentration of amygdalin increased; note β-glucosidase concentration was 0.1 mg/mL. The viability of HepG2 cells treated with L/D-amygdalin was statistically greater than those treated with D-amygdalin at every concentration tested. The IC_50_ of D and L/D-amygdalin was 6.37 mg/mL and 9.14 mg/mL, respectively (Figure 4B). In summary, the cytotoxic activity of amygdalin is reduced when isomerization occurs.

#### 2.2.2. Cell Morphology

**Figure 5:**
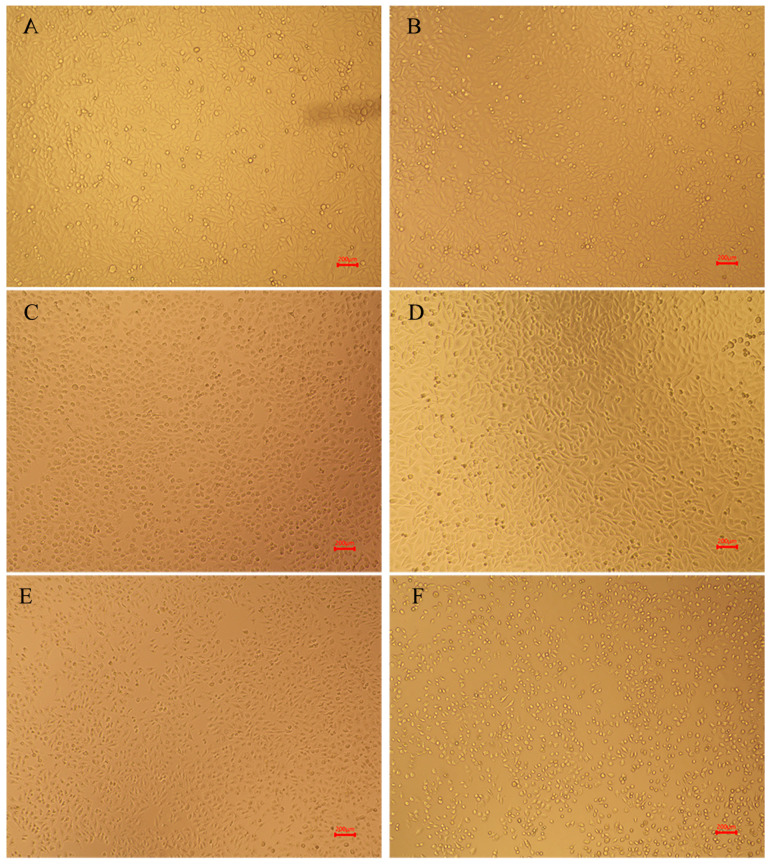
Morphology of HepG2 cells. (**A**) control group; (**B**) 6 mg/mL D-AMY + β-Glu; (**C**) 12 mg/mL D-AMY + β-Glu; (**D**) β-Glu; (**E**) 6 mg/mL L/D-AMY + β-Glu; (**F**) 12 mg/mL L/D-AMY + β-Glu.

The morphology of HepG2 cells incubated for 24 h with D- and L/D-amygdalin plus 0.1 mg/mL β-glucosidase is shown in Figure 5. Control HepG2 cells are irregularly polygonal, a typical tumor cell morphology, and are at a healthy density. The morphology of HepG2 cells incubated with 0.1 mg/mL β-glucosidase treatment was basically the same as those of the control group, indicating that β-glucosidase had no significant effect on HepG2 cell viability or growth. The morphology of cells incubated with 6 mg/mL of D-amygdalin changed from the irregular polygon to round, cell volume increased slightly, and cell density was significantly reduced relative to the control group. The reduction of cell mass is likely attributable to early stages of apoptosis [29]. Most of the cells treated with 6 mg/mL D/L-amygdalin were still irregular polygons, but the cell density had been significantly reduced compared to the control group. Cells incubated with 12 mg/mL D-amygdalin were completely ruptured; there was no integral cell structure; these cells were in the advanced stage of apoptosis. Cells in the 12 mg/mL D/L-amygdalin treatment group still had intact cellular structure. These results and the IC_50_ calculations demonstrate that D-amygdalin has significantly higher cytotoxic activity than L/D-amygdalin. Therefore, amygdalin should be prevented from isomerization during processing, transportation, and storage.

#### 2.2.3. Hydrolysis Rate of β-Glucosidase

**Figure 6:**
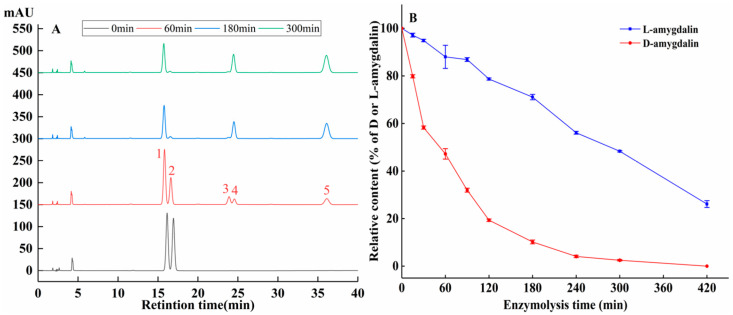
(**A**) HPLC of L- and D-amygdalin enzymolysis over time. 1: L-amygdalin; 2: D-amygdalin; 3: L-prunasin; 4: D-prunasin; 5: benzaldehyde. (**B**) Rate of enzymatic hydrolysis.

The reaction of β-glucosidase with amygdalin produces HCN, which has a key role in cytotoxic of activity of amygdalin. We analyzed the hydrolysis of D/L-amygdalin (isomer ratio was 1) by HPLC (Figure 6A). Enzymatic digestion of amygdalin is a multistep process [30]. First, the 1,4 glycosidic bonds at the end of amygdalin are broken, yielding a glucose molecule and prunasin. The prunasin molecule then yields a glucose molecule and mandelonitrile. Benzaldehyde and HCN are produced last.

As shown in Figure 6B, after 15 min of hydrolysis, the percentages of D and L-amygdalin remaining in the sample were 20.2% and 2.9% respectively. At 180 min, only 10.2% of D-amygdalin was un-hydrolyzed, while 71.1% of L-amygdalin was un-hydrolyzed. D-amygdalin was completely hydrolyzed at 420 min. The difference in the rate of hydrolysis of D- and L-amygdalin means a difference in the content of HCN and benzaldehyde produced during hydrolysis. This likely explains the difference in the growth and proliferation of HepG2 cells incubated with D- and L/D-amygdalin.

### 2.3. Preparation of Peach Kernel Amygdalin with a Low Isomer Ratio

#### 2.3.1. Solid to Liquid, Ethanol Concentration, Ultrasonic Power, and Temperature

**Figure 7:**
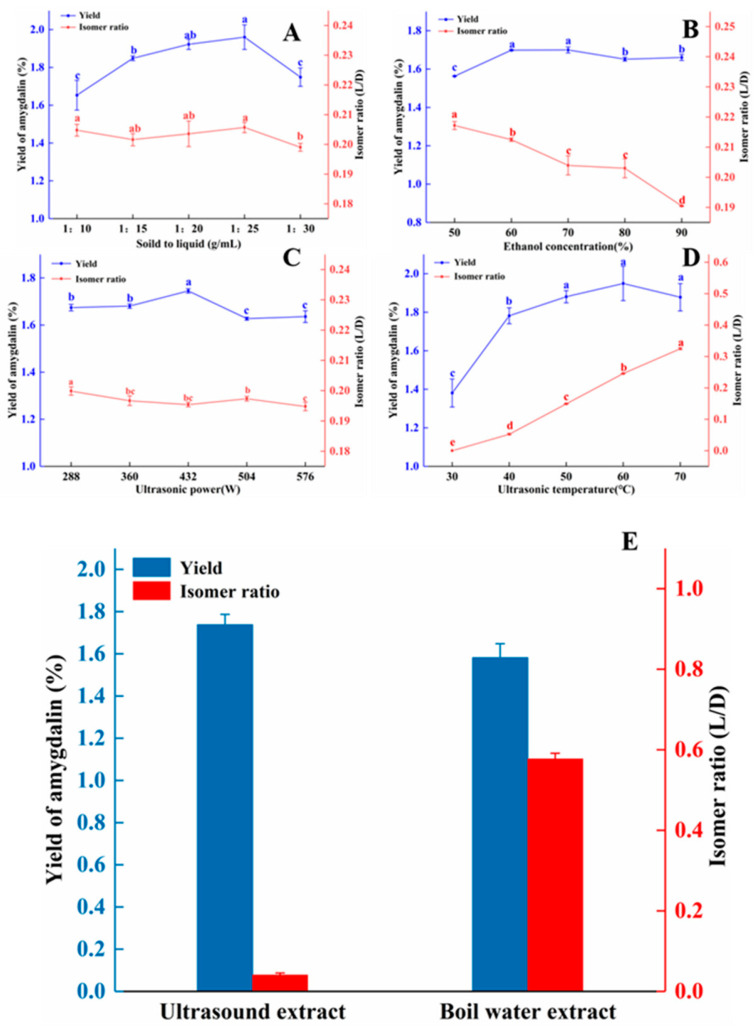
Single factor experiments: ultrasonic extraction parameters affect the yield and isomer ratio of amygdalin extracted from peach kernels. (**A**) solid-to-liquid ratio; (**B**) ethanol concentration; (**C**) ultrasonic power; (**D**) ultrasonic temperature. Different letters indicate significant differences (*p* < 0.05). (**E**) Comparison of amygdalin yield and isomer ratio from the ultrasonic and boiling water extraction methods. Note: ultrasonic extraction: solid to liquid was 1:25 (*w*/*v*), ultrasonic power was 432 W, ultrasonic temperature was 40 °C, and ethanol concentration was 80%. Boiling water extraction: solid to liquid was 1:25 (*w*/*v*), incubation temperature was 100 °C.

As demonstrated by the reduced cytotoxic activity of L/D-amygdalin, it is of great significance to avoid isomerization in the process of extraction the amygdalin from peach kernels to minimize the isomerization of amygdalin during the process of extraction. We tested the significance of solid to liquid ratio, ethanol concentration, ultrasonic power, and ultrasonic temperature through single factors experiments (Figure 7). Figure 7A shows the influence of solid to liquid (*w*/*v*) on the yield and isomer ratio; the yield of amygdalin from peach kernels increased as the solid-to-liquid ratio increased to 1:25 where it peaked; thereafter, yields declined. This may be due to the larger amount of solvent absorbing ultrasonic power [31]. There was no obvious correlation between the solid to liquid ratio and the isomerization of amygdalin.

The yield of amygdalin increased as concentration of ethanol increased from 50% to 60%, then remained steady or slightly decreased. The yield of amygdalin was 1.67% at the ethanol concentration of 70% (Figure 7B). This may be because the high concentration of ethanol promoted the dissolution of alcohol-soluble impurities in the peach kernels, thereby increasing the viscosity of the extraction solution and reducing mass transfer [32]. Consequently, the speed of amygdalin diffusion into the solvent was slowed, and yields declined. The isomer ratio of amygdalin decreased steadily with increasing ethanol concentration, as was also seen in Figure 1C.

As shown in Figure 7C, the yield of amygdalin increased somewhat as ultrasonic power increased to from 288 W to 432 W, then declined and held steady as the power increased to 576 W. This may be due to damage of cells walls by high Wattage and the leakage of large cell fragments into the solvent, resulting in a lower extraction efficiency [33]. With the increase in Wattage, the proportion of isomer rate showed a slight downward trend.

Figure 7D shows as the temperature increased during ultrasonication from 30 to 50 °C, the yield of amygdalin increased by just over half a percent; there was no significant change in yield as the temperature increased to 60 and 70 °C. With the increment of temperature, the sonochemical influents caused by the collapse of cavitation bubbles decreased, which could be due to increased vapor pressure and reduced surface tension. Temperature rising leads to a lowering of the viscosity of the solvent, which causes increased vapor pressure resulting in the formation of more bubbles. Moreover, due to the lesser pressure difference among the inner and outer side of the bubbles, they collapse with lesser intensity. This is also explained that ultrasound being less prominent at higher temperatures, and any advantage should not just be referred to by its effect [26,34]. Not unexpectedly, the isomer ratio increased with increasing temperature.

From these data (Supplementary Table S2), taking the yield and isomerization ratio of amygdalin as the index, the optimal parameters for extraction process are as follows: solid to liquid—1:25 (*w*/*v*); ethanol concentration—80%; ultrasonic power—432 W; and ultrasonic temperature—40 °C.

#### 2.3.2. Comparison of the Optimized vs. Common Extraction Process of Amygdalin

β-glucosidase, released from peach kernels by the crushing process, hydrolyzes amygdalin. To prevent this, the β-glucosidase in peach kernels need to be inactivated before extraction [35]. Commonly used methods for inactivating β-glucosidase include decoction, hot air, and microwave [36], all of which depend on the action of high temperature. Because high temperatures promote the isomerization of amygdalin Figure 2A, we investigated effect of these methods on the isomerization of amygdalin (Supplementary Figure S4). Among these, the boiling water method resulted in the greatest degree of isomerization, with an isomer ratio of 0.109; the ratios from the microwave and hot air methods were 0.063 and 0.034, respectively.

These results demonstrated that use of the hot air and microwave methods effectively avoids the isomerization of amygdalin in peach kernels under high temperature conditions. Compared with the hot air method, the microwave method not only requires a shorter time to extinguish β-glucosidase activity but also effectively avoids isomerization. Thus, we selected the microwave method to inactivate β-glucosidase.

To better illustrate the advantage of our optimized ultrasound method for extraction of amygdalin from peach kernels, we used the hot water extract method [37] (boiling water for 30 min) for comparison (Figure 7E). Using the ultrasound method, the yield of amygdalin was 1.76%. It is higher than boiling water extract (1.59%), but the isomer ratio was 0.04, significantly lower than with the boiling water method.

In summary, microwave treatment effectively inactivated β-glucosidase in peach kernels and maximized the retention of D-amygdalin. Ethanol ultrasonic assisted extraction of amygdalin from peach kernels, along with attention to temperature, and ethanol concentration resulted in good yields of D-amygdalin and high cytotoxic activity.

### 2.4. Stabilization and Vitro Release of D-Amygdalin

#### 2.4.1. Encapsulation Efficiency and Drug Loading Rate

Sodium alginate hydrogel was used to encapsulate the amygdalin for improving stabilization and utilization during process. As shows in Table 1, the encapsulation efficiency increased with the increasing of sodium alginate addition. While the addition of sodium alginate was 2% and further increased, the encapsulation efficiency remained constant and reached to be 85.93%. Meanwhile, the prepared hydrogel held the highest drug loading rate (19.21%).

#### 2.4.2. Thermal Stability

**Figure 8:**
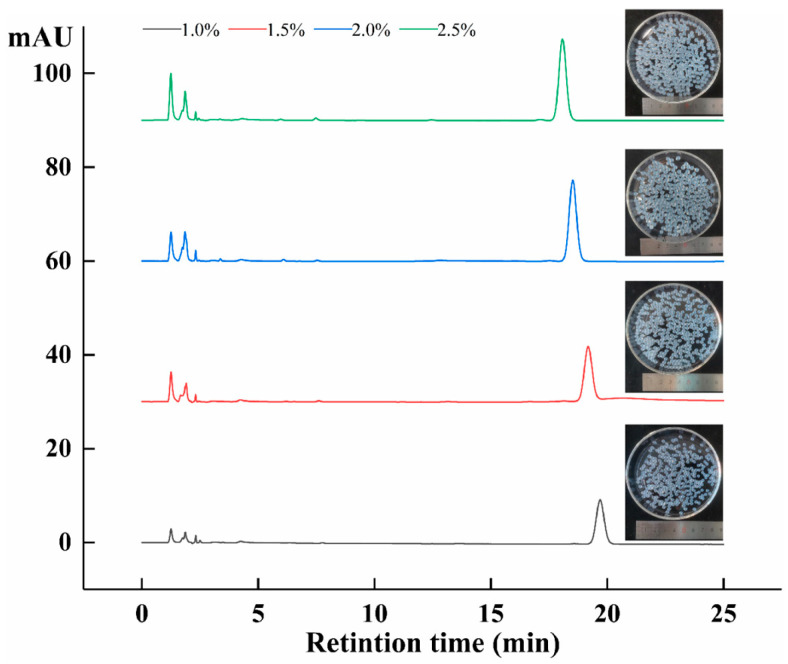
HPLC of different concentrations of sodium alginate–amygdalin hydrogel beads after heating at 80 °C.

When sodium alginate–amygdalin hydrogel beads were heated at 80 °C for 30 min, the isomerization of amygdalin was expressed as thermal stability. As shown in Figure 8, it can be seen that L-amygdalin was not detected in the four concentrations of hydrogel beads after heating. This indicates that the preparation of sodium alginate-loaded amygdalin hydrogel beads can effectively avoid the isomerization of amygdalin at high temperatures. It has been reported that alginate hydrogel beads can improve the thermal stability of some substances [38,39].

#### 2.4.3. FTIR Analysis

**Figure 9:**
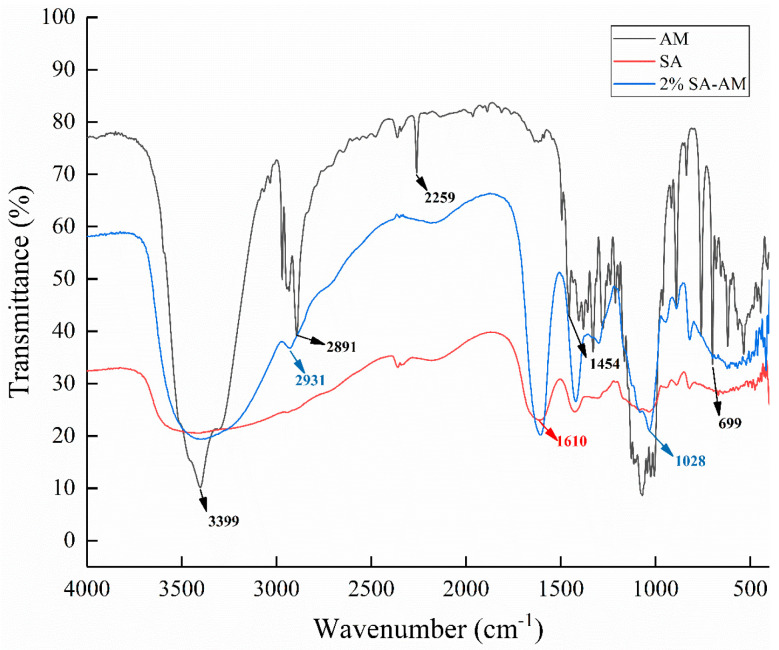
FTIR of amygdalin, sodium alginate, and 2% sodium alginate–amygdalin hydrogel beads.

The FTIR spectrum in Figure 9 shows that the characteristic peaks at 1610 cm^−1^ and 1407 cm^−1^ correspond to the stretching vibration of asymmetric and symmetric COO-carboxylate groups. The low-intensity characteristic band at 2259 cm^−1^ indicates that the nitrile group (–C≡N) has a low intensity. The strong vibration band at 1028 cm^−1^ (COC stretching) is attributed to the sugar structure of sodium alginate. In the spectra of sodium alginate–amygdalin hydrogel beads, the contraction vibrations at 699, 1028, and 2931 cm^−1^ basically coincided with the spectra of amygdalin [2]. This indicates that amygdalin was embedded in the interior of sodium alginate hydrogel beads.

#### 2.4.4. Swelling Behavior and Release In Vitro Digestion

**Figure 10:**
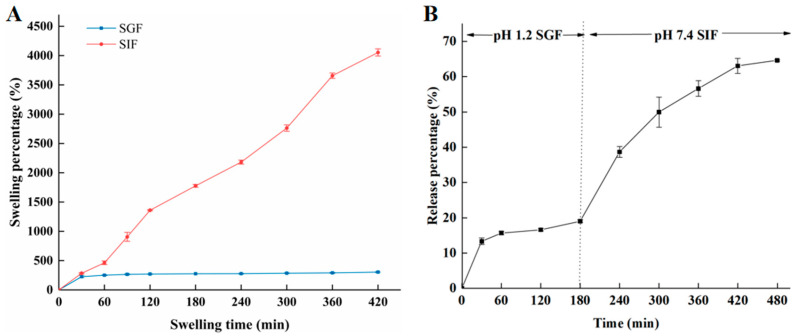
Swelling behavior (**A**) and release effect (**B**) of 2% sodium alginate–amygdalin hydrogel beads in vitro.

As shown in Figure 10A, after 420 min, the swelling percentage of hydrogel beads in SIF reached 4054%, while the swelling percentage in SGF was only 305%. This was mainly due to the fact that the strength of hydrogen bonding between COOH and OH groups from the alginate polymeric chain weakened in SIF solution. The COOH in the hydrogel beads were ionized into COO- [40]. Furthermore, the increase in COO- will produce electrostatic repulsion, disturbing the integrity of the hydrogel beads by loosening the structure of the hydrogel beads. Finally, more water molecules infiltrated into the beads. Compared with the swelling degree in SIF, the swelling degree in SGF is low. This may be due to conversion COO- into COOH groups under acidic condition [41]. On the other hand, in SGF solution, as shown in Figure 10B, the release of amygdalin was relatively slow, and the release rate was about 20% after reaching stabilization. In SIF solution, the release gradually increased during digestion; consequently, the release rate of amygdalin reached 64.65% at 480 min. The release effect in SIF was significantly higher than that in SGF solution, which was also consistent with the swelling behavior. During the release process in SIF solution, amygdalin was dissolved and then diffused through the swelling process [40]. The low swelling effect of SGF was obviously not conducive to the release of amygdalin. This indicates that the prepared amygdalin–sodium alginate hydrogel beads can sustain relative stability in SGF, and simultaneously, amygdalin could be released in the SIF, which was meaningful for adsorption and utilization.

## 3. Materials and Methods

### 3.1. Materials and Reagents

Amygdalin (Purity > 97%), was purchased from Macklin Biochemical Technology Co., Ltd. (Shanghai, China). β-glucosidase (≥4 U/mg) and acetonitrile (HPLC grade) were purchased from Sigma-Aldrich Co., (Tokyo, Japan). NaOH, HCl, Na_2_CO_3_, Na_2_HPO_4_, NaH_2_PO_4_, and CH_3_COONa were purchased from Sinopharm Chemical Reagent Co., Ltd. (Shanghai, China). DMEM high glucose medium (4.5 g/L), DMEM-F12 medium, and phosphate-buffered saline (PBS) were purchased from Corning Incorporated. Fetal bovine serum (FBS) was purchased from Gibco industries. 3-(4,5-Dimethylthiazol-2-yl)-2,5-diphenyltetrazolium bromide (MTT) and dimethyl sulfoxide (DMSO) were purchased from Solar Biochemical Technology Co., Ltd. (Beijing, China). Trypsin-EDTA Solution (0.25% trypsin) was purchased from Beyotime Biotechnology Co., Ltd. (Shanghai, China). Sodium alginate, KBr (FTIR grade > 99%), was purchased from Yuanye Biotechnology Co., Ltd. (Shanghai, China). Peach kernels were from ripened HuJing juicy peaches (Ningbo, China).

### 3.2. Analysis of Isomerization Factors

#### 3.2.1. Single Temperatures, Incubation Times, pH, and Ethanol Concentration

The single-factor conditions were as follows: incubation temperatures were 30, 40, 50, 60, 70, and 80 °C; times were 30, 60, 90, 120, and 150 min; pH of amygdalin solutions was adjusted to 2, 5, 7, 9, and 11, then placed at room temperature for 12 h; ethanol solutions were 20, 40, 60, 80, and 100% and incubated at 60 °C for 60 min. All reactions were performed in glass tubes.

#### 3.2.2. Container Material

A total of 1 mL amygdalin solution (0.5 mg/mL) was aliquoted to glass tubes (9 mm screw vial), plastic tubes (polypropylene), and stainless steel tubes (304 stainless steel) and then incubated at 60 °C for 60 min. Additionally, 10 mL of purified water was incubated at 80 °C for 30 min in glass tubes. The above solution was transferred to plastic tubes and incubated at 80 °C for 30 min. The isomer ratio of amygdalin was then determined. A total of 1 mL amygdalin solution (1 mg/mL) was added to 1 mL of purified water (control group), 0.01 M NaCl, CH_3_OONa, Na_2_HPO_4_, NaHCO_3_, and Na_2_SiO_3_. These mixtures were incubated at 80 °C for 30 min in plastic tubes. In addition, the effect of Na_2_SiO_3_ concentration (0.05, 0.5, 5, 50, and 500 μM) on isomerization was tested.

#### 3.2.3. The Combined Effect of Container Material, pH, and Temperature

Amygdalin (0.5 mg/mL) was dissolved in water which was adjusted to pH 11. The solution was then aliquoted into glass, plastic, and stainless steel tubes and incubated at room temperature for 12 h. For the interaction between pH and temperature, the amygdalin was dissolved in water, which was adjusted to pH 2, aliquoted into glass tubes and incubated at 30, 40, 50, 60, 70, and 80 °C for 60 min.

#### 3.2.4. Determination of Isomerization Ratios

Samples were analyzed using an Agilent 1260 HPLC (Agilent Technologies, Santa Clara, CA, USA), equipped with Variable Wavelength Detector (VWD) on an Agilent Poroshell 120 EC-C18 (4 μm 4.6 × 150 mm). The isomerization ratio of amygdalin was determined as described by Zhang et al. [35]. The isomer ratio of amygdalin was calculated according to the equation:

Isomer ratio = peak area_(L-amygdalin)_/peak area_(D-amygdalin)_ × 100%. (1)

### 3.3. Analysis of the Cytotoxic Activity of Amygdalin

Human hepatoma cells (HepG2 cells) were cultured in DMEM high glucose medium, supplemented with 10% inactivated FBS, 2 mmol/L L-glutamine, and 1% penicillin-streptomycin. Cells were maintained at 37 °C in a humidified 5% CO_2_ atmosphere; medium was replaced every two days until cells were confluent. The cytotoxic activity was assessed using an MTT assay [42]. Cells were seeded in 96-well plates at a density of 1 × 10^6^ cells/well and cultured for 12 h. 0.1 mg/mL β-glucosidase, and 2, 4, 6, 8, and 10 mg/mL of D-amygdalin or L/D-amygdalin (isomer ratio 1) were dissolved in DMEM medium and aliquoted onto cells. Cell viability was calculated using the following formula:

Cell viability (%) = (Treat-blank) OD570/(Control-blank) OD570 × 100%.(2)

The median effective doses (IC_50_) of D- and L/D-amygdalin were calculated using GraphPad Prism 8. The morphology of each treatment group was observed using an inverted fluorescence microscopy (Nikon ECLIPSE Ts2R-FL, Tokyo, Japan).

### 3.4. Hydrolysis of L/D Amygdalin

In plastic tubes, 900 μL of L/D-amygdalin (10 mg/mL, isomer ratio 1) was mixed with 100 μL of β-glucosidase solution (0.1 mg/mL) and incubated at 37 °C for 0, 15, 30, 60, 90, 120, 180, 240, 300, and 420 min. Samples were placed in a boiling water bath for 5 min to terminate the reaction then centrifuged at 12,000 rpm for 10 min. Supernatants were filtered through a 0.22 μm membrane, and the hydrolysis of L/D-amygdalin by β-glucosidase was analyzed using HPLC.

### 3.5. Extraction of Amygdalin from Peach Kernels

#### 3.5.1. Single Factor Extraction Experiment

Clean and unspoiled peach kernels were microwaved for 5 min at 400 W to inactivate β-glucosidase then ground using an IKA A11 Basic analytical mill (IKA Works Guangzhou, Guangzhou, China). The kernel powder was degreased using petroleum ether. Single-factor extraction conditions were as follows: solid to liquid ratios were 1:10, 1:15, 1:20, 1:25, and 1:30 (*w*/*v*). Ethanol concentrations were 50, 60, 70, 80, and 90%. Ultrasonic power was 288, 360, 432, 504, and 576 W and temperature was 30, 40, 50, 60, and 70 °C.

#### 3.5.2. Determination of Encapsulation Efficiency and Drug Loading Rate

A total of 100 mg of dried sodium alginate hydrogel beads were placed in a triangular flask. Then, 50 mL of PBS solution with pH 7.4 was added and shaken at 37 °C 120 rpm for 24 h. The weight of amygdalin was determined by HPLC, and the encapsulation efficiency and drug loading rate were calculated by the following equation [40]:

Encapsulation efficiency (%) = drug loading/theoretical loading × 100%(3)

Drug loading rate (%) = weight of drug in beads/weight of bead × 100%.(4)

#### 3.5.3. Orthogonal Experiments

Based on the single factor experiments, the orthogonal experiment of four factors and three levels was designed (see Table S1). The scores were calculated using the yield and isomer ratio of amygdalin:

Scores = (Yield/Yield_maximum_) × 50 + (Isomer ratio_minimum_/Isomer ratio) × 50. (5)

### 3.6. Encapsulation of Amygdalin

#### 3.6.1. Preparation of Alginate–Amygdalin Hydrogel Beads

According to the method of Camacho et al. [43], the sodium alginate of 200, 300, 400, and 500 mg was dissolved in 20 mL HCl solution with pH of 3.5. The gel was dropped into 0.1 M CaCl_2_ solution, preheated at 50 °C at a flow rate of 2 mL/min, then solidified for 60 min and further dried at room temperature for 24 h in a fume hood.

#### 3.6.2. FTIR Determination

All samples were ground into powder and fully mixed with KBr then compressed into wafers. FTIR (Nicolet iS50, Thermo Fisher Scientific Inc., Shanghai, China) was used to analyze samples at 400–4000 cm.

#### 3.6.3. Thermal Stability of Amygdalin

A total of 1 g of sodium hydrogel beads containing different concentrations of sodium alginate and 10 mL purified water was placed in glass bottle, which was heated at 80 °C for 30 min. The surface moisture of hydrogel beads was dried then grinded with 5 mL purified water and centrifuged at 12,000 rpm for 10 min to obtain supernatant. The isomerization of amygdalin was analyzed.

#### 3.6.4. Swelling Percentage

The swelling study was carried out using dried 2% alginate–amygdalin hydrogel beads, 0.01 M PBS (SIF, simulated intestinal fluid, pH 7.4) was used to simulate the colon environment, and HCl solution (SGF, simulated gastric juice, pH 1.2) was used to simulate the stomach environment. The dried hydrogel spheres were immersed in SIF and SGF at room temperature. After a predetermined interval of time, the swelling hydrogel beads were carefully separated from the solution, and the liquid on the surface of the hydrogel beads was sucked by the absorbent paper and weighed, and the swelling percentage of the hydrogel beads in different media was calculated.

#### 3.6.5. Release In Vitro Digestion

A total of 100 mg dried 2% sodium alginate–amygdalin hydrogel beads were added to 50 mL SGF solution and shaking incubation at 37 °C. All the hydrogel beads were taken out, and the residual water on the surface of the hydrogel beads was carefully absorbed with absorbent paper at 180 min. Then, all beads were transferred to a preheated 50 mL SIF solution and incubated in the same way.

### 3.7. Statistic Analysis

Data are presented as means ± SD and were analyzed using SPSS^©^ version 25. Data were compared using one-way ANOVA and individual differences were determined by Duncan’s test. A *p* value ≤ 0.05 was considered significant.

## 4. Conclusions

The isomerization of amygdalin is affected by temperature, pH, container material, and the water content of the solvent. Amygdalin begins to isomerize at 40 °C and at a pH of 9. SiO_3_^2−^, probably from glass during heating, promotes the isomerization of amygdalin at concentrations of 5 μM. Isomerization of amygdalin can be inhibited by using ethanol as a solvent, using an extraction solution with an acidic pH, and using plastic or stainless steel containers. As for cytotoxic activity, D-amygdalin shows higher cytotoxic activity than L/D-amygdalin. The optimum extraction parameters of amygdalin were obtained as follows: ultrasonic temperature—40 °C; ultrasonic power—432 W; solid to liquid—1:25 (*w*/*v*); and ethanol concentration—80%. Using these conditions, a higher yield of amygdalin (1.76%) with a lower isomerization ratio (0.04) can be obtained than by use of the common water extraction process. Finally, the hydrogel beads prepared by sodium alginate can avoid isomerization of amygdalin at high temperature (80 °C), sustain stabilization of amygdalin in the SGF, and gradually release in SIF. These data are helpful for preparing amygdalin with high cytotoxic activity.

## Figures and Tables

**Table 1:** The encapsulation efficiency, drug loading rate, and water content of different concentrations of sodium alginate–amygdalin hydrogel beads. Different letters indicate that there are significant differences (*p* < 0.05).

Alginate/%	Encapsulation Efficiency/%	Drug Loading Rate/%	Water Content/%
1.0	36.08 ± 2.09 ^c^	17.16 ± 0.20 ^c^	98.00 ± 0.17 ^a^
1.5	62.27 ± 3.09 ^b^	18.63 ± 0.18 ^b^	97.40 ± 0.09 ^b^
2.0	85.93 ± 2.05 ^a^	19.21 ± 0.10 ^a^	97.03 ± 0.10 ^c^
2.5	87.49 ± 0.99 ^a^	17.27 ± 0.06 ^c^	96.84 ± 0.08 ^c^

## Data Availability

The data that support the findings of this study are available from the corresponding author upon reasonable request.

## Supplementary Materials

The following supporting information can be downloaded at: https://www.mdpi.com/article/10.3390/molecules28114550/s1, Figure S1. Chemical structure of D-amygdalin and L-amygdalin. Figure S2. HPLC of amygdalin powder hot air heated in oven at 80 °C for 120 min. Figure S3. HPLC of amygdalin heating in plastic tube contained water which was heated in glass tube. Figure S4. Effect of enzymatic killing method on isomer ratio of amygdalin. Table S1. Orthogonal factors table. Table S2. The result of orthogonal experiment.

## Sample Availability

Samples of the compounds are available from the authors.
