# Supplementary material for: Isomerization and Stabilization of Amygdalin from Peach Kernels
Source: Molecules. 2023 Jun 5;28(11):4550. doi: 10.3390/molecules28114550 (PMC10254743; doi:10.3390/molecules28114550)
Supplement: Supplementary file 1 [file molecules-28-04550-s001.zip › molecules-2411433-supplementary.pdf]

Table S1. Orthogonal factors table

Level	Factors			
	Solid to liquid/(g/mL) A	Ethanol concentration/% B	Ultrasonic power/W C	Ultrasonic temperature/°C D
1	1:20	60	360	40
2	1:25	70	432	50
3	1:30	80	504	60

Table S2. the result of orthogonal experiment

Number	Factors				Yield （%）	Isomer ratio(L/D)	Scores
	A	B	C	D			
1	1	1	1	1	1.420	0.053	83.438
2	1	2	2	2	1.637	0.114	64.464
3	1	3	3	3	1.590	0.243	51.814
4	2	1	2	3	1.908	0.246	60.022
5	2	2	3	1	1.752	0.056	89.887
6	2	3	1	2	1.767	0.112	68.270
7	3	1	3	2	1.702	0.128	63.881
8	3	1	3	2	1.663	0.253	53.323
9	3	3	2	1	1.583	0.049	91.500
Scores k_1	66.950	69.491	68.721	88.652			
k_2	72.726	69.225	71.995	65.539			
k_3	69.568	70.528	68.527	55.053			
R	5.776	1.303	3.468	33.599			

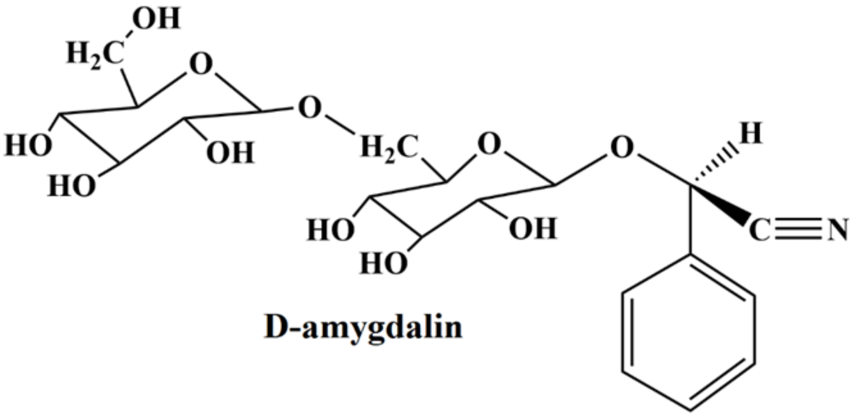

D-amygdalin

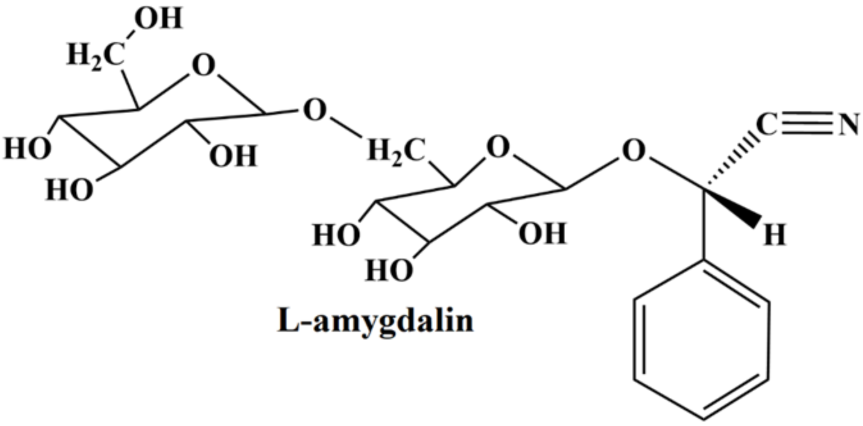

L-amygdalin

Figure S1. Chemical structure of D-amygdalin and L-amygdalin.

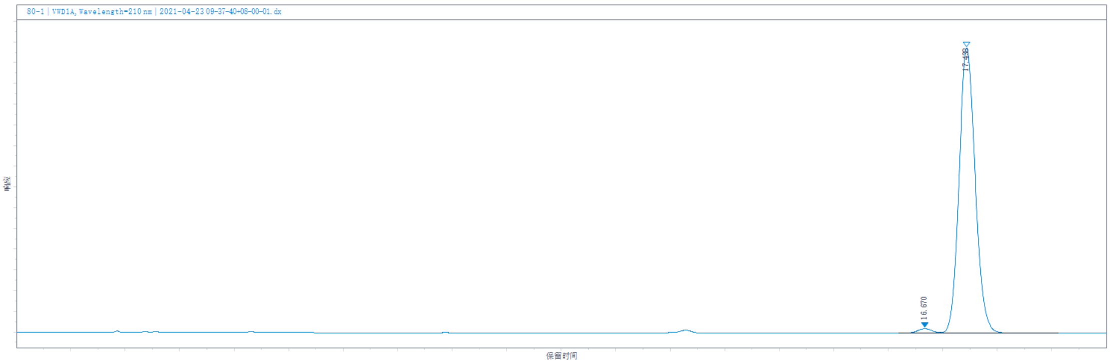

Figure S2. HPLC of amygdalin powder hot air heated in oven at 80 °C for 120 min

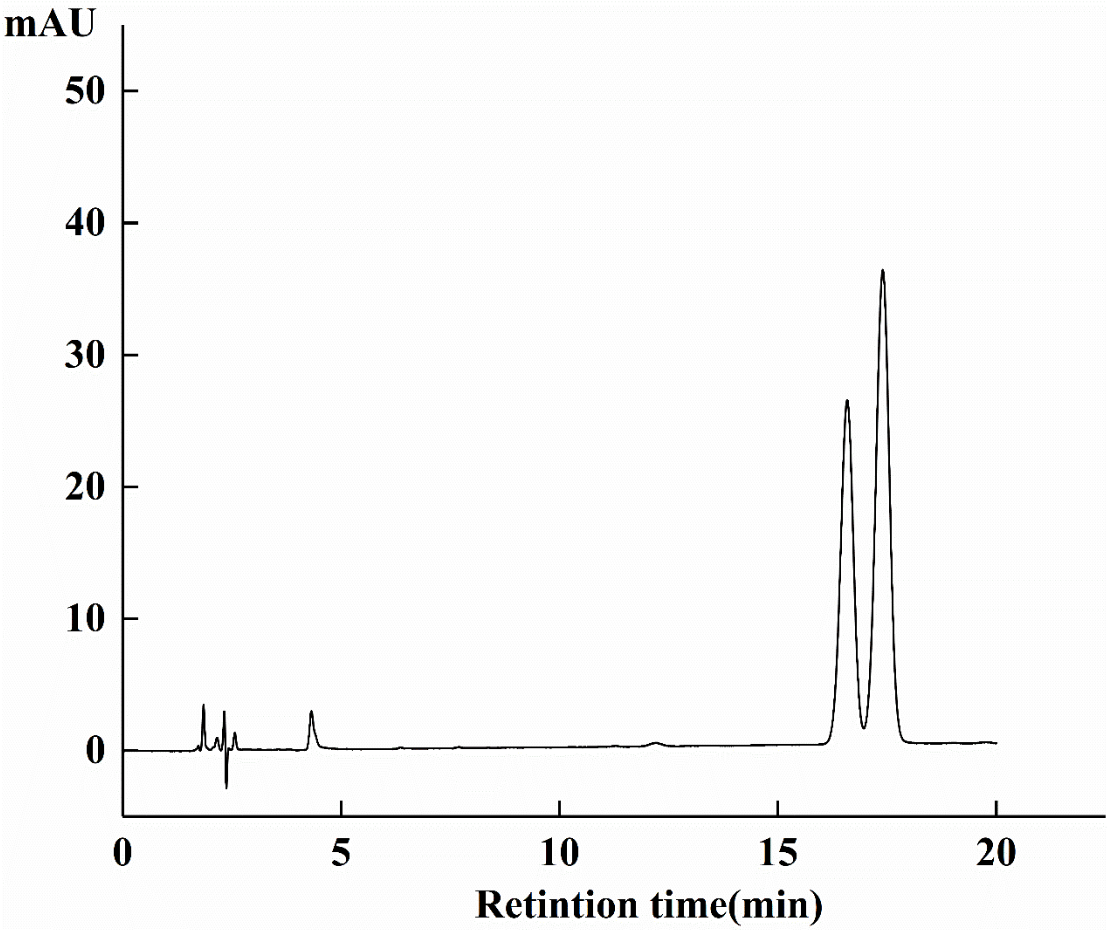

Figure S3. HPLC of amygdalin heating in plastic tube contained water which was heated in glass tube

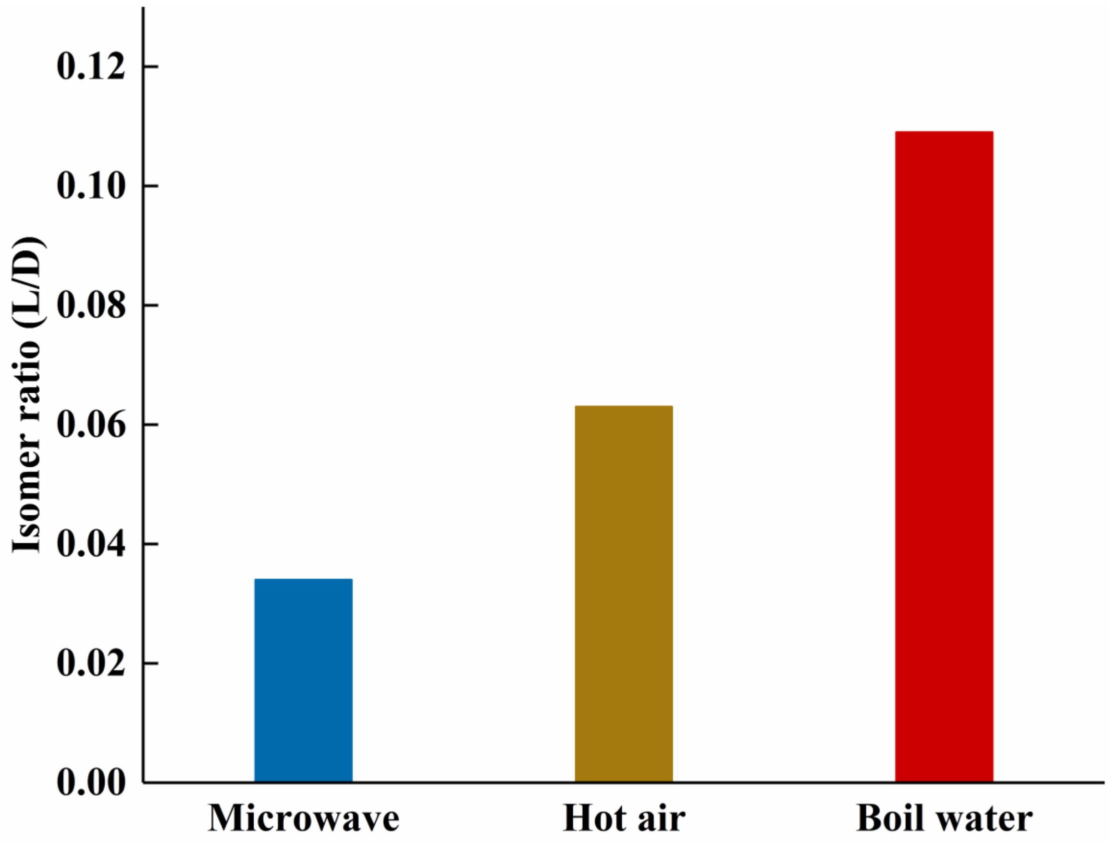

Figure S4. Effect of enzymatic killing method on isomer ratio of amygdalin
